# Selected Patients with Benign Prostatic Enlargement and Detrusor Underactivity may Benefit from Prostate Surgery: An Exploratory Study

**DOI:** 10.5152/tud.2026.26005

**Published:** 2026-06-16

**Authors:** Luís Vale, Ana Charrua, Carlos Martins- Silva, António Albino- Teixeira, Francisco Cruz, Tiago Antunes-Lopes

**Affiliations:** 1 Pharmacology and Therapeutics Unit, Department of Biomedicine, University of Porto Faculty of Medicine, Porto, Portugal; 2 Department of Urology, ULS São João, Porto, Portugal; 3 RISE-HEALTH, University of Porto Faculty of Medicine, Porto, Portugal; 4 RISE-Health, Department of Surgery and Physiology, University of Porto Faculty of Medicine, Porto, Portugal

**Keywords:** Benign prostatic enlargement, bladder contractility index, bladder voiding efficiency, detrusor underactivity, urinary ATP

## Abstract

**Objective:**

To evaluate non-invasive biomarkers of detrusor underactivity (DU) in men with benign prostatic enlargement (BPE) and their ability to predict clinical and urodynamic improvement following prostate surgery. Particular attention was given to urinary adenosine triphosphate (ATP) levels, bladder contractility index (BCI), and bladder voiding efficiency (BVE).

**Methods:**

This exploratory study included 24 men with BPE undergoing prostate surgery. Based on pre-operative pressure-flow studies (*P*/*F* studies), patients were divided into DU (BCI < 100) and non-DU (BCI ≥ 100) groups. Urinary ATP levels were compared among DU patients, non-DU patients, and age-matched healthy male volunteers. Clinical and urodynamic parameters, including BCI and BVE, were assessed before and 1 year after surgery.

**Results:**

Thirteen patients were classified as DU, and 11 as non-DU. Median urinary ATP levels did not differ between groups. One year after surgery, DU patients presented a significant improvement in International Prostate Symptom Score, quality of life, Qmax, and post-void residual volume. However, median BCI did not increase after surgery. Apart from BCI, all other parameters were similar between groups after surgery. In this cohort, all DU patients with a pre-operative BVE > 40% achieved surgical success, compared to <50% of those with BVE < 40%.

**Conclusion:**

Urinary ATP does not appear to be a useful DU biomarker in men with BPE. Although prostate surgery did not significantly improve detrusor function 1 year after surgery, most DU patients experienced clinical improvement. Pre-operative BVE seems a simple non-invasive tool to identify DU patients more likely to benefit from surgery.

**Main Points**

- Urinary adenosine triphosphate levels cannot differentiate men with benign prostatic enlargement and detrusor underactivity (DU) from those with normal detrusor function, precluding its utility as a non-invasive biomarker.
- After prostate surgery, most DU patients experience clinical and functional improvement.
- Bladder voiding efficiency >40% seems a valuable non-invasive predictor of surgical success in DU patients.

## Introduction

Chronic bladder outlet obstruction (BOO) due to benign prostatic enlargement (BPE) significantly disrupts bladder structure, ultimately leading to severe and potentially irreversible functional impairment.^1^ Though a commonly feared entity, detrusor underactivity (DU) lacks a precise definition.^2^^,^^3^ The International Continence Society defines DU as a urodynamic diagnosis, characterized by “low detrusor pressure or short detrusor contraction” in combination with a “low urine flow rate resulting in prolonged bladder emptying and/or a failure to achieve complete bladder emptying within a normal time span” (with a high post-void residual volume [PVR] potentially present).^4^ The bladder contractility index (BCI) has been used as an invasive measure of detrusor function, with poor contraction (DU) defined by less than 100.^5^ Recently, bladder voiding efficiency (BVE) was accepted as a non-invasive way of investigating DU in clinical practice, with normal values varying between 70% and 90% among studies.^6^

Preclinical animal models investigating DU showed that the bladder urothelium is implicated in early phases of DU development and in the recovery following prompt surgical relief of obstruction.^7^ Urothelial generation of adenosine triphosphate (ATP) plays a key role in both processes, with a decrease associated with DU and a recovery after deobstruction. As a matter of fact, urothelial ATP activates the afferent arm of the micturition reflex by binding P2X3 receptors in sub-urothelial nerves.^8^ However, in men, the importance of urothelial ATP in the development of DU and recovery of detrusor function after prostate surgery is uncertain. Additionally, whether urinary levels of ATP could be used as a non-invasive biomarker of DU in men with BPE is unclear.^9^

This exploratory study primarily aimed to evaluate whether urinary ATP levels could predict the presence of DU in men with BPE prior to prostate surgery, in parallel with BCI and BVE. The effect of surgery on these parameters, as well as on clinical and urodynamic variables, were evaluated 1 year after the procedure in 2 groups of patients: 1 with DU and another with normal detrusor function. It was anticipated that urinary ATP in these patients followed the same pattern seen in rodents and that patients with DU had poorer urodynamic parameters 1 year after prostate surgery.

## Material and Methods

### Study Population

This was an exploratory study and no a priori sample size calculation was performed due to the absence of preliminary clinical data. The enrollment period was between September 2017 and September 2020. Twenty-four men with BPE were recruited from the outpatient clinic and proposed for prostate surgery. To ensure a BPE etiology, exclusion criteria included alternative etiologies for male lower urinary tract symptoms, such as neurologic impairment, history of pelvic trauma/surgery/irradiation, diagnosis of diabetes for more than 10 years, acute urinary tract infection within 1-month, interstitial cystitis, and bladder or prostate cancer. Patients with renal insufficiency (values of creatinine 2 times above the normal limit) were also excluded. Patients had moderate to severe symptoms, defined as an International Prostate Symptoms Score (IPSS) ≥ 8. Demographic data, IPSS, quality of life (QOL) questionnaires, and renal function tests were obtained from all patients. Also, an ultrasound scan evaluated prostate volume, PVR and assessed signs of hydronephrosis.

The study was conducted in accordance with ethical standards and approved by the local ethics committee (Protocol No. 332-16, September 25, 2017). All informed consent was obtained from the subjects.

### Bladder Contractility Index and Bladder Voiding Efficiency

Detrusor underactivity was investigated using a pressure-flow (*P*/*F*) study, where Pdet.Qmax is the detrusor pressure at maximum flow rate and Qmax is the maximum flow rate. The DU was defined using BCI [BCI = Pdet.Qmax + 5Qmax]. Patients with BCI < 100 were assigned to the DU group, whereas those with BCI ≥ 100 were assigned to the non-DU group. From *P*/*F* studies, PVR volume and BVE [BVE (%) = (pre-micturition volume (mL) − PVR (mL)) × 100/pre-micturition volume (mL)] were also determined. The studies were performed in both groups before and 1 year after the surgery.

### Adenosine Triphosphate Measurement

Before initiating the *P*/*F* study, urine was collected and stored at −80°C until further analysis. Urine samples from age-matched volunteers, which were used as controls, were collected and stored for posterior analysis similarly. Urinary ATP levels were quantified by luminometry using ATP BIOLUMINESCENCE ASSAY KIT HSII (Sigma, Portugal), using the standard addition method, following the manufacturer’s protocol. Baseline urinary ATP levels of DU, non-DU patients and age-matched asymptomatic male healthy volunteers (control group) were compared. Urinary creatinine measurements were not available for these samples.

### Prostate Surgery and Surgical Success

Prostate surgical treatments included transurethral resection of the prostate (TURP) or enucleation. The percentage of TURP in the DU and non-DU group was 85% and 91%, respectively (Table 1.). One year after surgery, invited for a new consultation in which clinical evaluation and *P*/*F* study were repeated, urine was collected and stored for repeated ATP analysis. Surgical success was defined by voiding without catheter and BVE ≥ 80%. The BVE values below 80% are commonly used in the literature to indicate incomplete bladder emptying, whereas higher values are generally considered compatible with adequate voiding efficiency.^6^ Clinical parameters (age, prostate volume, IPSS and QoL) and functional data (Qmax, Pdet.Qmax, BCI, BOOI, PVR, and BVE) were also evaluated to assess clinical and further functional recovery following prostate surgery.

## Statistics

Non-parametric tests were used to reduce false positive outcomes. A *P *value of less than .05 was considered statistically significant. At baseline, median urinary ATP levels of all groups were compared using the Kruskal–Wallis test. To compare baseline characteristics of DU and non-DU groups, Mann–Whitney test was used for continuous variables and the Likelihood Ratio test for categorical variables. To compare pre- and post-surgical outcomes of the DU group, the Wilcoxon signed-rank test was used. Surgical success predictors were analyzed using the Mann–Whitney test. Apart from urinary ATP analyses (GraphPad Prism Version 10.0.1 for Windows, GraphPad Software, Boston, Massachusetts USA, www.graphpad.com), all statistical tests were performed using IBM SPSS Statistics for Windows, version 26 (IBM Corp., Armonk, N.Y., USA).

## Results

### Baseline Bladder Contractility Index and Bladder Voiding Efficiency

According to BCI < 100 or ≥ 100, 13 patients were included in the DU group and 11 in the non-DU group, respectively. The baseline clinical and urodynamic evaluation of DU and non-DU groups is summarized in Table 1.

At baseline, the DU group, compared to the non-DU group, presented lower BCI (56 [38, 88] vs. 134 [110, 152], *P* < .001) and lower BVE (45 [12, 61] vs. 92 [74, 100] %, *P* = .001). The median PVR in the DU group was significantly higher than in the non-DU group, as well as the use of bladder catheter (5 patients in the DU group needed permanent catheter). The DU group also presented significantly lower Qmax and Pdet.Qmax than the non-DU group. All other baseline variables were similar between the groups.

### Adenosine Triphosphate Results

**Figure 1.**
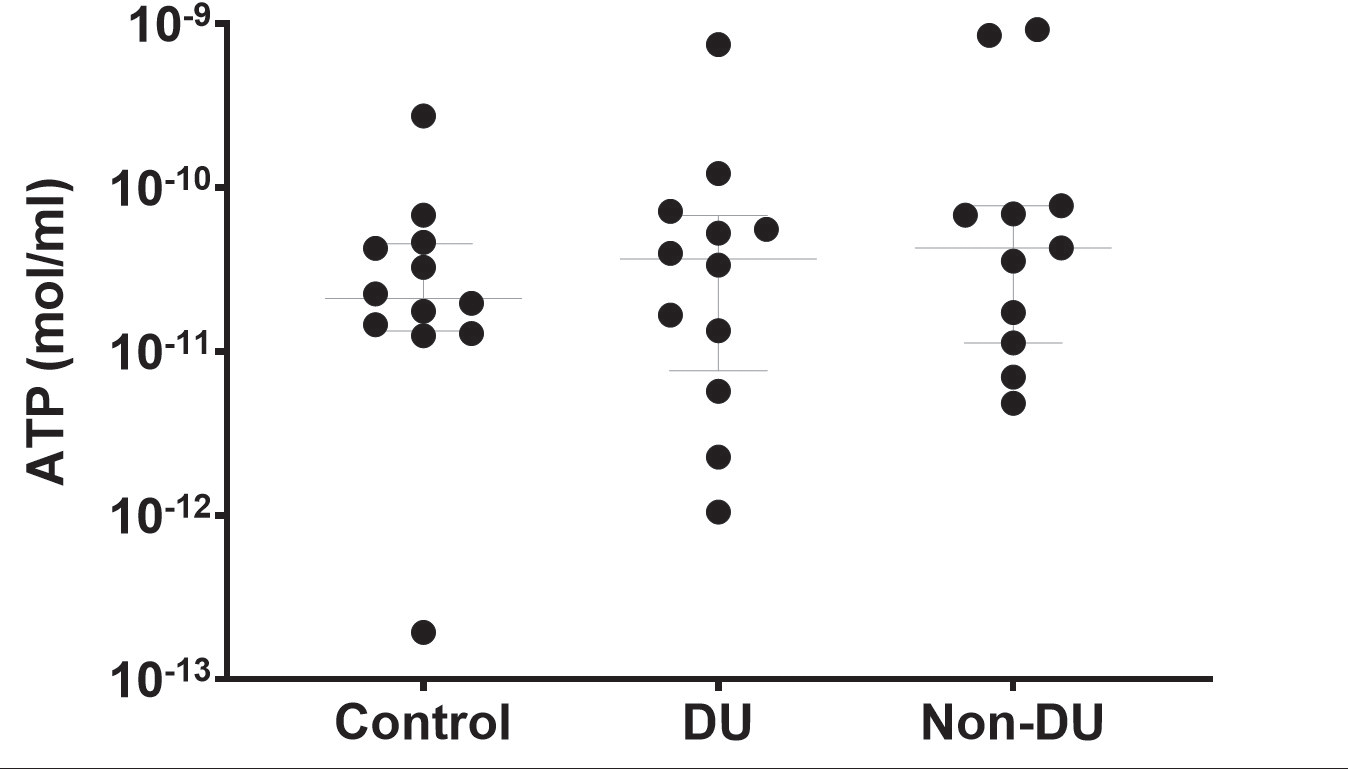
Comparison of urinary ATP levels between DU, non-DU patients, and healthy controls. Scatter plot depicting urinary ATP levels (mol/mL) in 3 groups: healthy controls (n = 12), DU patients (n = 12), and non-DU patients (n = 11). The median ATP levels are represented by the horizontal lines within each group. No significant differences in urinary ATP levels were observed between the groups (*P* > .05).

The median urinary ATP levels of DU (n = 12), non-DU patients (n = 11), and healthy volunteers (n = 12) were 3.7 × 10^−11^, 4.3 × 10^−11^ and 2.1 × 10^−11^ mol/mL, respectively (Figure 1). These values were statistically similar (*P* = .612). Given these findings, the ATP of urine collected from patients after prostate surgery was not analyzed, as no utility was expected and samples were eliminated.

### Clinical and Urodynamic Parameters of Detrusor Underactivity and Non-Detrusor Underactivity Patients 1 Year After Surgery

Clinical and *P*/*F* evaluation of the DU group pre- and 1 year post-surgery are summarized in Table 2. Post-surgery, there was a significant improvement in IPSS and QoL scores. Additionally, BVE, Qmax, and PVR had a marked significant improvement. Interestingly, 9 out of 12 patients (75%) presented BVE ≥ 80% post-surgery. Although there was a slight increase in median BCI, it was not statistically significant (56 [38, 88] vs. 79 [49, 86], *P* = .202). Actually, 1 year after surgery, only 2 out of 12 DU patients (17%) presented a normal BCI ≥ 100. One patient in the DU group still needed a bladder catheter, while all the non-DU patients emptied the bladder without aid.

Table 3 compares the clinical and urodynamic outcomes of 12 DU and 8 non-DU patients who accepted to repeat the invasive urodynamics 1 year after surgery. Clinical data captured by IPSS and QoL scores were identical. Qmax, PVR, and BVE were also identical between the 2 groups. Only BCI in the DU group was significantly lower than in the non-DU group.

A univariate analysis of clinical and functional parameters was performed, aiming at identifying predictors of surgical success in DU patients (Table 4). Pre-operative BVE emerged as the only parameter able to predict surgical success. In this small cohort, all patients with a BVE above 40% before surgery experienced surgical success. In contrast, only 2 out of 5 patients with pre-operative BVE below 40% had surgical success.

## Discussion

Adenosine triphosphate proved to be a good candidate in preclinical models.^7^ However, the clinical study did not find a significant difference in urinary ATP levels among DU patients, non-DU patients, and healthy volunteers, leading us to conclude that urinary ATP is not a useful biomarker for the diagnosis of DU in men. These results contrast with preclinical findings where urothelial ATP was strongly correlated with bladder contractions in female rats with partial BOO.^7^ This discrepancy may stem from physiological differences between animals and humans.^10^ Nevertheless, it should be noted that previous clinical data have suggested ATP as a marker for several benign bladder conditions, such as DU, overactive bladder (OAB), and compensated BOO. Krishnan et al^11^ reported a small but significant difference in urinary ATP levels of DU and healthy male volunteers. Silva-Ramos et al^12^ found a positive correlation between urinary ATP and voided volume in men with BOO and detrusor overactivity. In contrast, in a randomized placebo-controlled study in women with OAB, the P2X3 antagonist Eliapixant did not meet the primary efficacy endpoint, which evaluated the change in the mean number of urgency urinary incontinence episodes per 24 hours, nor the secondary endpoints related to storage symptoms.^13^ All these data indicate that, in men, urothelial ATP does not play a prominent role in initiating voiding contractions.

In addition, it might be relevant to recall that detrusor contraction in rats has a strong purinergic component, through ATP release from cholinergic nerves, while this purinergic component is not relevant in humans.^3^ Thus, taking into consideration all these inconsistent findings and the complex method to measure urinary ATP, one should conclude that ATP, on the contrary to the initial hypothesis, lacks characteristics of a human biomarker for predicting DU. Of note, although normalization to urinary creatinine is frequently used to account for urine dilution, ATP concentrations were reported as absolute values. This approach ensured methodological consistency with the preclinical studies that generated the study hypothesis and used the same luminometric assay.^7^ Furthermore, creatinine measurement and ATP quantification had to be performed in different independent laboratories, which precluded creatinine normalization in the present study.

One year after surgery, significant improvements were observed in IPSS, QoL scores, and several urodynamic parameters of DU patients, as evidenced by improvements in Qmax, reduction in PVR, and increase in BVE. Together with the fact that most DU patients on indwelling catheter could empty the bladder spontaneously after surgery, it appears there is potential for functional recovery, as also demonstrated in other cohorts.^14^^,^^15^ Naturally, one may ask if the observed improvement stemmed from enhanced detrusor function, decrease in outlet resistance, or both. For instance, Pdet.Qmax improved, although not significantly, from a median of 33 to 22 cmH_2_O. Also, BCI, which was used as criteria for the definition of DU in this study, did not increase after surgery. This raises concerns about the capacity of myocytes to recover once injured by chronic obstruction. Therefore, functional recovery seen in this study (and possibly in other studies of DU patients after prostate surgery) should be mostly attributed to the decrease in outlet resistance. In accordance, Qmax, PVR, and BVE of DU patients had a good functional recovery, similar to the non-DU patients.

From a clinical point of view, IPSS and QoL were quite similar between DU and non-DU patients 1 year after surgery, suggesting that prostate surgery is a good alternative to improve symptoms in DU patients with BPE.

Considering that no utility of urinary ATP in predicting DU could be demonstrated and that these patients had the potential to recover detrusor function after prostate surgery, it was investigated whether any urodynamic parameters could help identify patients with DU who might benefit from surgery.

Currently, DU diagnosis relies on invasive urodynamic studies, an invasive procedure.^4^ Consequently, BVE has gained attention as a potential non-invasive marker for DU.^6^ In this cohort, all DU patients with pre-operative BVE > 40% achieved surgical success. In contrast, the outcome of DU patients with a BVE below 40% was not predictable. Finding a cut-off value of BVE may be an important aid in pre-operative counseling. If validated in larger studies, this simple and non-invasive parameter could help guide pre-operative counseling and shared decision-making in men with DU considering prostate surgery. It is suggested that a BVE above 40% before prostate surgery in DU patients should be further investigated as an indicator of surgical success.

One limitation of the study is the small sample size, which might affect the generalization of the results. In addition, the limited sample size may have reduced the statistical power of the study. Therefore, the absence of statistically significant differences between groups in some postoperative parameters should not be interpreted as proof of equivalence and may reflect a Type II error. In this context, the findings regarding BVE should be regarded as observations that require validation in large-scale, multicenter, prospective studies. Another limitation lies in the heterogeneity of the DU group, which included both patients who voided spontaneously and those with chronic urinary retention requiring an indwelling catheter—the most severe form of DU. Furthermore, the unequal dropout rate between groups after surgery (8% in the DU group versus 27% in the non-DU group) may have introduced bias favoring the DU group. Recruiting non-DU patients willing to repeat urodynamic studies 1 year after surgery proved particularly difficult. Even DU patients who were voiding satisfactorily were often reluctant to repeat invasive testing. Consequently, the incomplete follow-up with invasive urodynamics may introduce selection bias and should be considered when interpreting postoperative urodynamic outcomes.

Nevertheless, future studies with larger cohorts and long-term follow-up periods should be considered to investigate the fate of myocytes in patients who had the diagnosis of DU before prostate surgery.

In conclusion, while urinary ATP is not a reliable DU biomarker in men with BPE, the findings suggest that pre-operative BVE above 40% may represent a useful non-invasive tool to identify DU patients more likely to benefit from prostate surgery. Even though prostate surgery does not seem to improve detrusor function, the study showed that DU patients had clinical improvement after surgery.

## Figures and Tables

**Table 1.** Baseline Clinical and Urodynamic Characteristics of DU and Non-DU Groups

	Median (Q1, Q3) or n (%)		*P*
	DU	Non-DU	
Clinical variable			
Patients (n)	13	11	–
Age (Years)	71 (68, 77)	69 (59, 72)	.252
IPSS	23 (19, 23)	18 (13, 23)	.203
QoL	4 (4, 5)	4 (3, 5)	.710
Prostate volume (mL)	50 (37, 71)	54 (47, 67)	.569
Creatinine (mg/dL)	0.83 (0.77, 0.90)	0.77 (0.70, 1.0)	.693
Hydronephrosis (%)	2 (15)	1 (9)	.639*
Bladder catheter (%)	5 (38)	1 (9)	.085*
TURP (%)	11 (85)	10 (91)	.639*
Urodynamic Variable			
BCI	56 (38, 88)	134 (110, 152)	<.001
BVE (%)	45 (12, 61)	92 (74, 100)	.001
Qmax (mL/s)	4.0 (1.9, 8.6)	10.0 (6.8, 13.0)	.004
Pdet.Qmax (cmH_2_O)	33 (25, 40)	74 (40, 122)	<.001
PVR (mL)	231 (125, 386)	34 (0, 116)	.002

Statistical significance (*P*) was determined using the Mann-Whitney test for continuous variables and the Likelihood Ratio test for categorical variables (*). Significant differences are highlighted.

BCI, bladder contractility index; BPE, benign prostatic enlargement; BVE, bladder voiding efficiency; DU, detrusor underactivity; IPSS, International Prostate Symptom Score; non-DU, non-detrusor underactivity; Pdet.Qmax, detrusor pressure at maximum flow rate; PVR, post-void residual volume; Qmax, maximum urinary flow rate; QoL, quality of life; TURP, transurethral resection of the prostate.

**Table 2.** Clinical and Urodynamic Outcomes of Prostate Surgery in DU Group

Variable	Median (Q1, Q3) or n (%) of DU Patients		*P*
	Pre-Op	1 year Post-Op	
Patients (n)	13	12	–
IPSS	23 (19, 23)	7 (3, 10)	.005
QoL	4 (4, 5)	1 (1, 2)	.005
Qmax (mL/s)	4.0 (1.9, 8.6)	11.9 (5.0, 14.8)	.009
Pdet.Qmax (cmH_2_O)	33 (25, 40)	22 (20, 30)	.097
BCI	56 (38, 88)	79 (49, 86)	.202
PVR (mL)	231 (125, 386)	20 (0, 133)	.003
BVE (%)	45 (12, 61)	96 (8, 100)	.017

The table shows the changes in clinical and urodynamic parameters 1 year after prostate surgery. Values are expressed as median [Q1, Q3]. Statistical significance (*P*) was determined using the Wilcoxon signed-rank test. Significant differences are highlighted.

BCI, bladder contractility index; BVE, bladder voiding efficiency; DU, detrusor underactivity; IPSS, International Prostate Symptom Score; Pdet.Qmax, detrusor pressure at maximum flow rate; PVR, post-void residual volume; Qmax, maximum urinary flow rate; QoL, quality of life.

**Table 3.** Clinical and Urodynamic Outcomes of Prostate Surgery in DU and Non-DU Groups

Variable	Median [Q1, Q3]		*P*
	DU Group 1 Year Post-Op	Non-DU Group 1 Year post-op	
Patients (n)	12	8	–
IPSS	7 (3, 10)	4 (2, 10)	.515
QoL	1 (1, 2)	1 (0, 2)	.762
Qmax (mL/s)	11.9 (5.0, 14.8)	19.5 (9.1, 25.0)	.246
BCI	79 (49, 86)	155 (84, 180)	.003
PVR (mL)	20 (0, 133)	0 (0, 165)	.536
BVE (%)	96 (8, 100)	100 (49, 100)	.375

The table shows the changes in clinical and urodynamic parameters 1 year after prostate surgery. Variables include International Prostate Symptom Score (IPSS), quality of life (QoL), maximum urinary flow rate (Qmax), bladder contractility index (BCI), post-void residual volume (PVR) and bladder voiding efficiency (BVE). Values are expressed as median [Q1, Q3]. Statistical significance (*P*) was determined using the Mann-Whitney test. Significant differences are highlighted.

**Table 4.** Predictors of Surgical Success in DU Group

Variable	Surgical Sucess?		*P*
	No	Yes	
Patients (n)	3	9	–
Bladder catheter (n) (%)	2 (67)	3 (33)	–
Age (Years)	68 (19)	71 (68, 75)	.578
Prostate volume (mL)	40 (31)	50 (39, 69)	.644
IPSS	23 (5)	23 (22, 24)	.622
Qmax (mL/s)	0.8 (12.0)	4.0 (3.1, 7.1)	.354
Pdet.Qmax (cmH_2_O)	21 (22)	33 (29, 40)	.266
BCI	25 (82)	56 (53, 80)	.517
BOOI	15 (6)	26 (12, 33)	.354
PVR (mL)	448 (731)	231 (106, 302)	.116
BVE (%)	1 (20)	45 (27, 72)	.036

The table presents univariate analysis of pre-operative clinical and urodynamic parameters to identify predictors of surgical success in DU patients. Parameters include age, bladder catheter, prostate volume, International Prostate Symptom Score (IPSS), maximum urinary flow rate (Qmax), detrusor pressure at maximum flow rate (Pdet.Qmax), bladder contractility index (BCI), bladder outlet obstruction index (BOOI), post-void residual volume (PVR), and bladder voiding efficiency (BVE). Values are presented as median [Q1, Q3], except for the subgroup without surgical success (n = 3), where values are presented as median (range) due to the small sample size. Statistical significance (*P*) was calculated using the Mann–Whitney *U*-test. Significant differences are highlighted.

## Data Availability

The data that support the findings of this study are available on request from the corresponding author.
